# Report on the Sanitary Condition of Chicago, and Prevalence of Diseases, from April 1, to August 1, 1865

**Published:** 1865-09

**Authors:** N. S. Davis

**Affiliations:** Chicago, Ill.


### ARTICLE XXXII.

### REPORT ON THE SANITARY CONDITION OF CHICAGO, AND PREVALENCE OF DISEASES, FROM APRIL 1ST, TO AUGUST 1ST, 1865.

BY N. S. DAVIS, M.D., and Prof., Chicago, Ill.

Read to the Chicago Medical Society, August 4th, 1865.

The three Spring months of this year were, as a whole, more pleasant than the average for a series of years. There was less rain, the soil and roads became settled and dry earlier, and there were fewer sudden and extreme atmospheric changes. And, though the streets and alleys abounded in filth, as usual, yet there had been, during the winter and spring, less of the refuse of distilleries, slaughtering and rendering houses, &c., allowed to enter the Chicago River, and, consequently, it had been much less offensive than during the same part of the two preceding years. The last half of May and the first week in June, were unusually dry, so much so, as to cause apprehension of injury to the growth of agricultural products. The air, however, was cool and pleasant. From the 15th to the 25th of June, the atmospheric temperature was higher than the average for that part of the month. Showers of rain fell almost every morning, and the afternoons were clear, hot, and oppressive; the atmosphere being nearly filled with aqueous vapor, and the wind blowing lightly from the south and south-west.

During this time, the ordinary diarrhœas and cholera-morbus of Summer commenced, especially among children, and on the morning of the 24th, I was called to two adults, presenting all the symptoms of epidemic cholera. One was a man, on Rush Street, in the North Division, and the other was a female, on South Clark Street, near the river. The rice-water discharges, the small, feeble pulse, the sunken eyes, the husky voice, and the muscular cramps, were all as fully exhibited as in the majority of cases of cholera, when that disease was epidemic in the Summer of 1854. During the same day I also saw three severe cases of dysentery.

On the morning of the 25th, the wind changed to the north-west, accompanied by showers and a cool bracing air. It continued cold and dry until the 28th, during which all the new attacks of bowel-complaints, whether in children or adults, that came under my observation, presented the symptoms of dysentery.

On the morning of the 28th, some rain fell, the wind changed to the south, and the afternoon was warm and oppressive. From this to the 7th of July, the winds continued from the south and south-west, with occasional showers and a high temperature. The high temperature and high degree of atmospheric moisture rendered the influence oppressive and relaxing to the animal economy. During this period, the attacks of serous diarrhœa, cholera-infantum, and dysentery again increased rapidly, and a few cases were of sufficient severity to present a strong resemblance to epidemic cholera.

On the morning of the 7th of July, the wind suddenly changed to the north, and blew cold, followed by rain in the evening. From that time to the 1st of August, the weather continued cool and rainy, with a predominence of winds from the north and north-east. During the whole time, there were not three consecutive days of clear, dry, and hot weather, such as we usually have in the middle and latter part of July. During the sixteen years that I have lived in this city, I have not known such a predominance of cold and rain in the month of July. Soon after the change to cold, on the 7th of July, the attacks of serous diarrhœa and cholera-morbus became less frequent, while those of dysentery became more numerous, and were often complicated with catarrhal symptoms, as cough and soreness in the chest. During the last half of July, not less than 12 cases of severe asthma, mostly connected with bronchial irritation, came under my care; which is much more than the usual number for that season of the year.

Most of the cases of diarrhœa and dysentery have been readily controlled by proper remedies, and, hence, the ratio of mortality has not been high. I have observed but little tendency to fevers of any type. Hence, the four months included in this report, have presented an aggregate mortality below the average for the last three years. This will be apparent from the following statistics derived from the monthly report of the Health Officer:—

| Health Officer:— | 1864. | 1865. |
|---|---|---|
| Mortality in April, | 298 | 256 |
| " May, | 309 | 229 |
| " June, | 262 | 195 |
| " July, | 426 | 426 |
| Total, | 1295 | 1106 |
| Excess in four months of 1864, | | 189 |

When it is remembered that the population of the city during the four months of 1865 was several thousand more than during the corresponding months of 1864, the difference in the ratio of mortality will be very striking.

It may be well to remark that the first thorough saturation of the Chicago River with the offal and refuse of the slaughter-ng-houses, rendering establishments, distilleries, hog-yards, &c., occurred in the Autumn of 1863. It was then, for the first time, that the atmosphere of the whole city became offensively impregnated with the putrid effluvia from both branches of the river, and from the neighborhood of Bridgeport. And not only so, but the hydrant water supplied to the inhabitants became impregnated with the same materials.

The first unusual prevalence of disease noted in connection with the sanitary conditions just alluded to, was an extensive and severe epidemic of erysipelas, which began in the Fall of 1863, and continued until the Spring of 1864. Not long after the commencement of the erysipelas epidemic, continued fevers were found to be more prevalent and severe than usual. Many of the cases presented all the characteristics of true typhus. The prevalence and fatality of these fevers were much above the average of previous years, during all the first three quarters of 1864. Another disease which added materially to the mortality of the last quarter of 1863, and the first half of 1864, was small-pox, which was both extraordinarily prevalent and fatal. Indeed, this disease did not cease its unusual prevalence until the Spring of 1865. The ratio of mortality in Chicago, for 1864, was much above the average for a series of years, and much higher than for any previous year since the epidemic of cholera, in 1854. A detailed examination will show that this excess of mortality in 1864, as well as in the last half of 1863, was owing, principally, to the unusual prevalence of erysipelas, continued fevers, and small-pox.

The extraordinary stench which pervaded the atmosphere of the city, and impregnated the water, during the Autumn of 1863, and the succeeding Winter, induced the city authorities to bury the large heaps of putrid offal that had been deposited on the prairie in the vicinity of the south-west part of the city, and to adopt more stringent measures for preventing similar accumulations in future, either on the prairie or in the river. The consequence has been that, during the first six months of the present year, neither the atmosphere nor the water of the city has been so offensive as during the previous eighteen months. Coincidently with this improvement in the condition the of atmosphere and water, so far as relates to the impregnation of putrid animal matter, we find a striking diminution in the ratio of mortality, as shown by the foregoing statistics. It is also apparent, on examining the details of the monthly reports, that the low ratio of mortality for the last four months, is mostly owing to the diminished prevalence of erysipelas, continued fevers, and small-pox.

Thus, in April, 1865, the number of deaths from erysipelas was 2, continued fevers 12, and small-pox 4; in May, from erysipelas 3, continued fevers 8, and small-pox 5; in June, from erysipelas 1, continued fevers 7, and small-pox 3; in July, erysipelas 1, continued fevers 15, and small-pox 0. In the corresponding months of 1864 the deaths from erysipelas and continued fevers were more than double the above figures, while from small-pox they amounted to an aggregate of 100 or more than six times the number indicated by the above figures. We have thus in the comparison of the mortality of the city during the two years past, in connection with the well-known impurities of the air and water from the presence of putrid animal matter, another striking illustration of the direct influence of such matters on the prevalence and mortality of some of the most important diseases of the zymotic class.

In speaking of the atmospheric conditions and changes, during the last four months, it was stated that during a week of warm, damp, and sultry weather between the 18th and 25th of June, attacks of diarrhœa and cholera-morbus first became frequent; and that during a week of still warmer and more oppressive weather, embracing the first seven days of July, similar attacks of bowel-affections became still more frequent and severe, some of them presenting every symptom of true cholera. The unusual cold and wet that prevailed nearly all the rest of the month of July, was attended by fewer cases of serous diarrhœa and cholera-morbus, but a larger prevalence of dysentery. The progress of these affections, and their rapid increase during the month of July, is shown in the monthly report of mortality by the Health Officer. In his table for April, we find the following: dysentery 0, diarrhœa 2, teething 4, cramps 19; for May, dysentery 2, diarrhœa 9, teething 6, cramps 13; for June, dysentery 1, diarrhœa 13, teething 6, cramps 12; for July, dysentery 21, diarrhœa 120, teething 22, cramps 35. What the Health Officer means by the word "*cramps*" in his report, I am wholly at a loss to conceive. It will be seen that it stands responsible for no less than 79 deaths during the last four months; 35 of which occurred during the month of July. That it is not used as synonymous with convulsions, is evident from the fact that the latter term also appears in each monthly table, covering as many deaths as could be reasonably supposed to occur from convulsive diseases. That some of the cases reported under the head of *cramps* were simply cases of cholera-morbus accompanied by muscular cramps, especially during the month of July, is highly probable, but how many, it is impossible to state, and hence they cannot be properly included in the class of bowel-affections. If we exclude these and include those attributed to *teething*, the whole number of deaths from dysentery, cholera-morbus, and diarrhœa in each of the four months will be as follows:—

| | | | | |
|---|---|---|---|---|
| In April, | Dysentery, | 0 | Diarrhœa, | 6 |
| May, | do | 2 | do | 15 |
| June, | do | 1 | do | 19 |
| July, | do | 21 | do | 142 |

Thus while the gross mortality for July is more than double that of June, three-fourths of the increase is attributable directly to bowel-affections; and that too chiefly in young children. For instance, in June, of a gross mortality amounting to 195, only 77 were children under five years of age. In July, out of a gross mortality of 426, there were under 5 years of age 271, or considerably more than one-half of the whole number. It is a common opinion, both in and out of the profession, that a large part of the summer mortality among children arises from eating unwholesome vegetables and fruit. This is certainly not sustained by the Health Officer's figures. Thus, during the past month of July, while 271 deaths occurred in children under

5 years of age, only 14 occurred in children between 5 and 10 years. Yet those of the latter age in this city certainly consume a much larger quantity and variety of vegetables and fruit than those of the former. The figures taken in another aspect equally refute the more popular and far more mischievous idea, that most of the fatal bowel-affections of young children are owing to "*teething.*" It is quite certain that the number of children undergoing the process called *teething*, in the month of July, did not differ materially from the number undergoing the same process during either of the three preceding months. And yet the infant mortality for these several months is stated as follows:—

| | | | | | | |
|---|---|---|---|---|---|---|
| Deaths of children | under 5 years | of age | in | | April,----- | 102 |
| do | do | " | " | " | May, ----- | 91 |
| do | do | " | " | " | June,----- | 77 |
| do | do | " | " | " | July, ----- | 271 |

The refutation will be still more complete if we place in this connection the mortality directly from bowel-affections.

| | | | | | | | |
|---|---|---|---|---|---|---|---|
| Whole number | of deaths | from | Bowel-affections | in | | April,- | 6 |
| " | " | " | " | " | " | May,- | 17 |
| " | " | " | " | " | " | June,- | 20 |
| " | " | " | " | " | " | July,- | 163 |

More than fifteen years of close and careful observation in this city, has satisfied me that the prevalence and fatality of dysentery, diarrhœa, and cholera-morbus among children, bears no relation either to the consumption of vegetables and fruit, or to the process of teething; but is determined almost wholly by atmospheric conditions. During the whole fifteen years, I have not known a single week of continuous high temperature, with the prevailing winds from the south or south-west, occuring between the middle of June and the last of August, that was not accompanied by a marked increase in the number and severity of the attacks of cholera-morbus and diarrhœa in young children. If to the continuous high temperature and southerly winds, is added an excess of atmospheric moisture and the usual emanations from densely populated cities, the number and fatality of the attacks will be still further increased. But I have already extended this report to an undue length, and will call your attention to only one more topic, and that is, the very imperfect and unsatisfactory statistics of mortality furnished by the Health Officer of this city. It is probable that the gross mortality for each month is given correctly, as well as the mortality during the several periods of life; but the statement of the *causes* of *death*, is wholly unreliable, and much of it unintelligible. Thus in the table for July, we have assigned as *causes* of deaths the following: cold 1, cramps 35, childbirth 6, dropsy 8, liver-complaint 1, summer-complaint 94, spasm 1, teething 22, tumor 1, &c. Are we to infer that the individual dying from cold, *froze* to death; or did he die from bronchitis, pneumonia, tonsilitis, or something else? Where were the *cramps* that killed 35 of our citizens in one month, and what was the disease that gave rise to them? Did the 6 who died from *childbirth*, actually die from obstructions or difficulty in the delivery or from some one of the diseases incident to confinement? Were the 8 who died from *dropsy* affected with organic disease of the heart, kidneys, liver, ovaries, or what? Then the 22 victims to teething. Are we to infer that they actually became fatally exhausted from the natural growth of the teeth? If not what was the real disease from which they died? The 94 cases attributed to *summer-complaint*, were undoubtedly bowel-affections; but how many of them were dysentery, how many simple diarrhœa, and how many cholera-morbus? The worthlessness of such reports needs no comments to make it apparent.

